# A case study of local ecological knowledge of shellfishers about edible cockle (*Cerastoderma edule*) in the Ria de Aveiro lagoon, Western Iberia

**DOI:** 10.1186/s13002-022-00507-x

**Published:** 2022-03-05

**Authors:** Heitor O. Braga, Ulisses M. Azeiteiro, Luísa Magalhães

**Affiliations:** 1 CESAM - Centre for Environmental and Marine Studies, Department of Biology, University of Aveiro, 3810-193 Aveiro, Portugal; 2 CAPES Foundation, Ministry of Education of Brazil (BEX: 8926/13-1), Caixa Postal 250, Brasília, DF 70040-020 Brazil

## Abstract

**Background:**

The cockle is available to numerous fishing villages in Europe, especially Portugal. In the Ria de Aveiro, there is a lack of a fisheries management program and the need for new ecological studies on cockle biology, ecology, and conservation. We shared local ecological knowledge (LEK) highlights about the cockle—*Cerastoderma edule* (Linnaeus 1758) in the Ria de Aveiro in favor of adaptive management of this bioresource.

**Methods:**

Semi-structured interviews with sixty shellfishers in this coastal lagoon were carried out during April and May 2021. LEK data on the biology and ecology of the cockle were analyzed using an ethical-emic approach and the model of integration of different individual skills. These informal data were compared with previously published data for the species, the Fish Base, and GBIF databases.

**Results and discussion:**

The average minimum size of the cockle for capture was 23.4 mm, and the average capture per tide was 137.12 kg. The areas with the highest productivity and the most shellfish were RIAV1 and RIAV2. Cockles inhabit areas of sand and mud at an average depth of 2.71 cm. Feeds are mainly small particles, plankton, mud, and algae. The main predators were crabs, European plaice, and bird species. Cockles spawn primarily in late spring and summer. As of 2010, there was a slight decrease in cockle stocks in the Ria de Aveiro due to overfishing, increased rainfall, and changes in the sediment. Considering and analyzing this knowledge is essential for a better understanding of the environmental context the cockles thrive in the view of users of the natural resource.

**Conclusion:**

Informal data shared by shellfishers in the Ria de Aveiro were typical of filter-feeding bivalves. LEK may assist in planning future management plans for cockles, and unrefuted data may serve as untestable hypotheses. Ethnobiological studies in the Ria de Aveiro lagoon with other species may improve the management of this system since multiple fisheries are carried out in this coastal area.

**Supplementary Information:**

The online version contains supplementary material available at 10.1186/s13002-022-00507-x.

## Background

The edible cockle (Mollusca: Cardiidae)—*Cerastoderma edule* (Linnaeus 1758) is a native, infaunal siphonate, and filter feeder bivalve [1, 2]. It occurs in subtidal and intertidal zones in sandy bays and estuaries in coastal areas around the northeast Atlantic from Norway to Morocco and across the Baltic, Mediterranean, and Black Sea [3, 4]. It prefers sites with a higher salinity gradient located near downstream [5]. This euryhaline bivalve species have external fertilization, with high fecundity rates and dispersal potential due to the pelagic larval stage [6]. It is engineer species, as it physically disturbs the water column and the sediments, allowing the presence of microphytobenthos in the ecosystem [7]. *Cerastoderma edule* also plays a crucial role in ecosystem services [8], as a food source for bird species [9, 10], as carbon storage in the form of CaCO3 [11], as bioindicator species [12], and as a link between trophic levels in the food web [7].

This marine bivalve is among the most targeted bivalves in Europe, where they play a crucial socio-economic and cultural role in fishing villages [13, 14]. In Portugal, the volume of catches in 2020 showed an increase in the record of cockles (+ 44.5%), resulting in a greater weight in the total volume of bivalve caught [15]. Cockles are also hugely relevant for fishing and aquaculture in the Ria de Aveiro coastal lagoon [16]. The capture of this bivalve in the Aveiro coastal lagoon can exceed 1000 tons per year [17]. Emphasizing that even with sales higher than those declared at fishing auction, cockle capture represented about 92% of landings (about 3,500 tons of *C. edule*) and about 85% of total revenue in 2018 at Ria de Aveiro (about 4 million Euros) [18].

Nonetheless, there is still a gap in ethnobiological studies on mollusks in Central Portugal (Iberian Peninsula), especially regarding cockles (*C. edule*) in the Ria de Aveiro coastal lagoon. There is only one recent cockle study that used the fishers’ LEK to share the changes in cockle fishing over the last few decades, as well as the impact of the COVID-19 pandemic on this artisanal fishery [19]. It is also observed that there is no specific management plan for the cockle [18], despite the sociobiological role and economic interest of the species in the region [20, 21]. Establishing an efficient coastal management program in the Aveiro region faces several obstacles, mainly due to the scarcity of biological data on the cockle [18].

Exploring the intrinsic knowledge that communities dependent on biological resources possess can be a crucial support tool for further conservation actions [22]. This type of approach, if well planned and executed, can manage insights and contribute to the structuring of a more sustainable and applicable community-based management [23]. LEK-based methods can also be necessary for formulating and implementing fisheries-related policies and rules when local people cooperate and participate in the management process [24].

A more in-depth study of the benefits of bivalves (such as cockles) contributes to promoting this coastal bioresource through a more socio-ecological management practice with the involvement of local villages [25]. Thus, this investigation aimed to document ethnobiological data of the edible cockle—*C. edule* (Linnaeus, 1758) in one of the most relevant biodiversity hotspots in the west of the Iberian Peninsula. This LEK approach shared informal data from the Aveiro fishing villages on habitat, predators and prey, food items, spawning period, and ethnoconservation. We also obtained information on artisanal cockle fisheries through the LEK of shellfish collectors to support the cockle databases in favor of a future structuring of a management plan for this bivalve in the Ria de Aveiro coastal lagoon, Portugal.

## Methods

### Study site

**Fig. 1:**
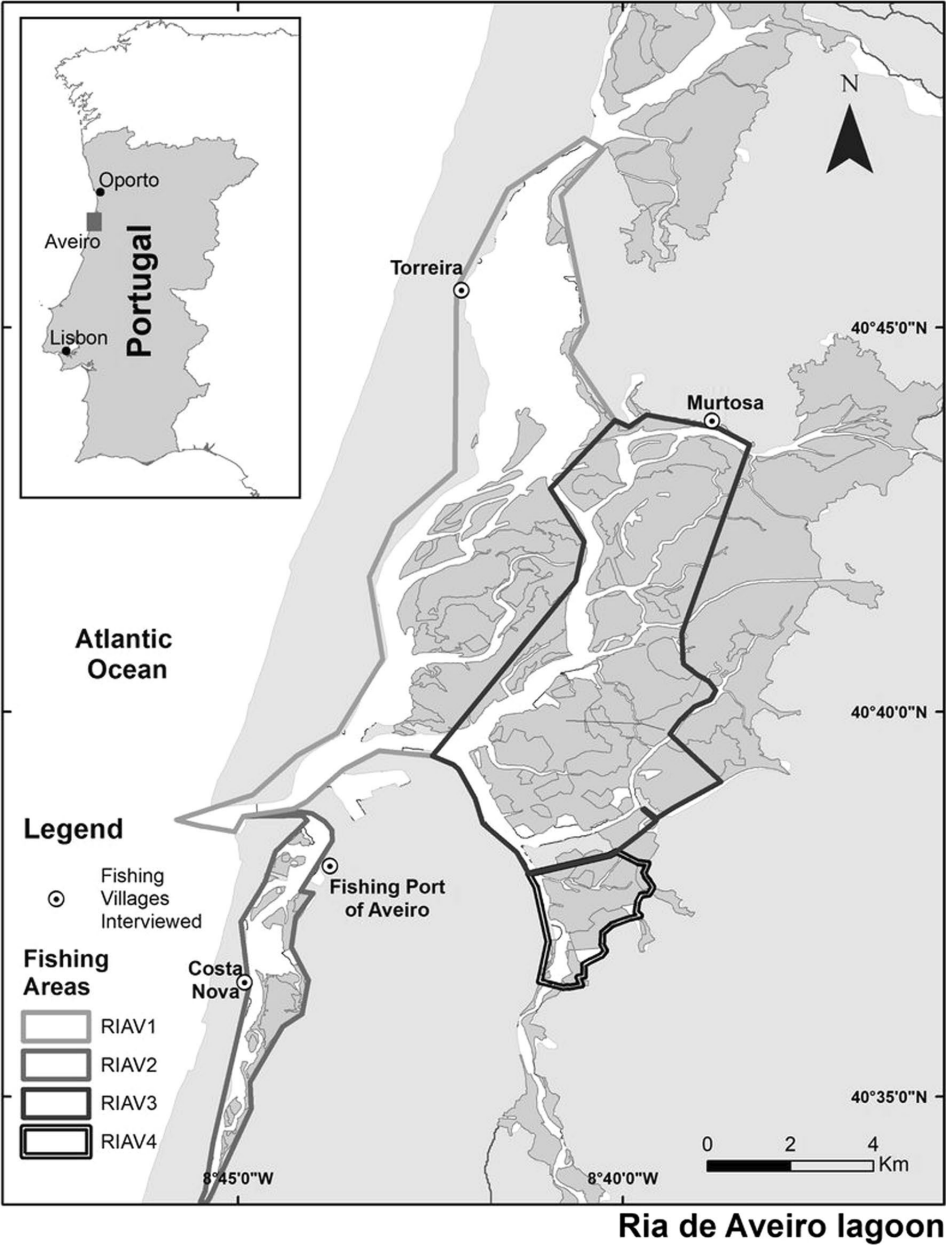
Map showing the critical fishing villages where this ethnobiological study was carried out and the distribution of bivalve production zones in the Ria de Aveiro lagoon (RIAV1, RIAV2, RIAV3, and RIAV4) in the District of Aveiro, Portugal. *Source*: Correia, S.

The Ria de Aveiro coastal lagoon is located predominantly in the District of Aveiro on the northwest coast of Portugal (4° 38′ N, 8° 44′ W; Fig. 1). This mesotidal coastal lagoon is a shallow, temperate, and well-mixed system and is considered one of the most extensive continuous salt marshes in Europe [26, 27]. The mouth of the lagoon is artificially maintained, has an average depth of 1 m, except in the navigation channels, where it can vary from 7 to 20 m [28]. The Ria de Aveiro lagoon is constituted by four main channels with several branches forming inner basins, mudflats, and small islands [29]. The crucial channels of the Ria are S. Jacinto-Ovar, Mira, Ílhavo, and Espinheiro [17]. This vertically homogeneous lagoon has a width and length of about 10 km and 45 km, with an area of 66 km^2^ at low tide and 82 km^2^ at high tide [30]. The central freshwater flows into this multiestuarine ecosystem come from the Vouga and Antuã rivers (≈ 70%) [31].

The Ria de Aveiro has been divided into four bivalve production areas by competent authorities on the mainland of Portugal (Dispatch No. 1851/2017, of March 3, 2017) [32]. The classification of estuarine-lagoon zones and their respective production areas in Aveiro are RIAV1 (Triangle of Currents—Moacha), RIAV2 (Ria de Aveiro, Mira Channel), RIAV3 (Ria de Aveiro, Main Channel—Espinheiro) and RIAV4 (Ria de Aveiro, Ílhavo Channel). This estuarine environment was classified as one of the protected areas of Natura 2000 [20]. It is part of a Special Protection Area (SPA) at the European level to guarantee existence and conservation of the most valuable and threatened habitats and species in Europe [33]. Considered valuable natural capital, the Ria has also been listed in the EU Birds Directive (79/109/CEE), EU Habitats Directive and an International Long-Term Ecosystem Research (ILTER Network) site [34]. In addition to multispecific fisheries, this coastal lagoon supports salt production, bait digging, sports activities and tourism [35]. The Aveiro lagoon has also a rich socio-cultural heritage that requires more sustainable management policies, mainly due to the various pressures that can alter this rich ecological and natural legacy [36].

### Shellfishers and harvesting areas

Cockle shellfishers were sampled in four areas in the Ria de Aveiro lagoon, Aveiro District, Portugal (Fig. 1). We highlighted the landing point at Costa Nova Beach, the Fishing Harbour of Aveiro, Abrigo Port (Torreira), and Abrigo do Bico Port (Murtosa). The essential fishing associations in this area are the Association of Artisanal Fishing of the Region of Aveiro (APARA) and the VianaPesca Producers Organization (VianaPesca O.P). In APARA, about 134 shellfish collectors (multispecific bivalve catchers) were registered in 2020, and in the O.P of VianaPesca, there were about 279 in 2021.

### Sampling protocol

Semi-structured interviews [37] were applied to shellfishers that had some relationship with cockle harvesting in this coastal lagoon. The sampling was opportunistic with shellfishers who would point out possible respondents at the main landing points in the Ria de Aveiro with the support of the smartwatch Huawei GT2 Pro-29F (v.11.0.5.22). This electronic device indicated real-time tidal change, allowing a greater probability of finding shellfishers at the fishing landing points. Interviews were conducted individually by the responsible researcher (HOB) collaborating with a trained resident biologist who belonged to one of the traditional fishing villages of Aveiro. Fieldwork trips were carried out daily in the morning and the afternoon. Successive daily visits to sampling points and adding resident data collectors often create a more friendly and trustworthy atmosphere in interviews [38]. This intercultural collaboration also generated multiple opportunities for transferring traditional knowledge and new scenarios for future scientific studies in these villages [39]. We used several desirable guidelines for an ethnobiologist in Methods and Techniques in Ethnobiology and Ethnoecology to maintain high ethical and scientific standards during field campaigns [40].

We guaranteed the interviewee's anonymity and explained the research objectives in detail before the interview takes place. We then delivered an informed consent (IC) with general survey information and institutional data. The ethical guidelines suggested by the International Society of Ethnobiology were followed in this study [41]. All the recommendations of Portugal’s Directorate-General for Health (DGS) were duly respected due to the current SARS-CoV-2 coronavirus pandemic (responsible for the disease COVID-19). The questionnaire applied contained open-ended issues [42] about the profile of the shellfishers, cockle fisheries, and local ecological knowledge (LEK) related to the habitat, predators, prey, spawning, and the ethnoconservation of the edible cockle (*Cerastoderma edule*) in the Ria de Aveiro lagoon (Additional file 1).

### Data analyses

The shellfishers’ knowledge was categorized and systematized by topics in Microsoft Excel for Microsoft 365 MSO. Fieldwork approached an emic-ethical distinction [43], following a native subject perspective and a researcher-observer perspective. The model of integrating different individual skills was used to analyze the qualitative data made available, which takes into account all the information made available in the data collection [44]. This set of informal knowledge was quantified in citations. The species data already published confronted this informal ecological knowledge when necessary [45]. The biological name of the species presented in the study followed the Global Information System on Fishes (Fish Base version 06/2021) [46] and the Global Biodiversity Information Facility (GBIF) [47].

## Results and discussion

### Shellfishers’ knowledge about cockle fisheries

**Fig. 2:**
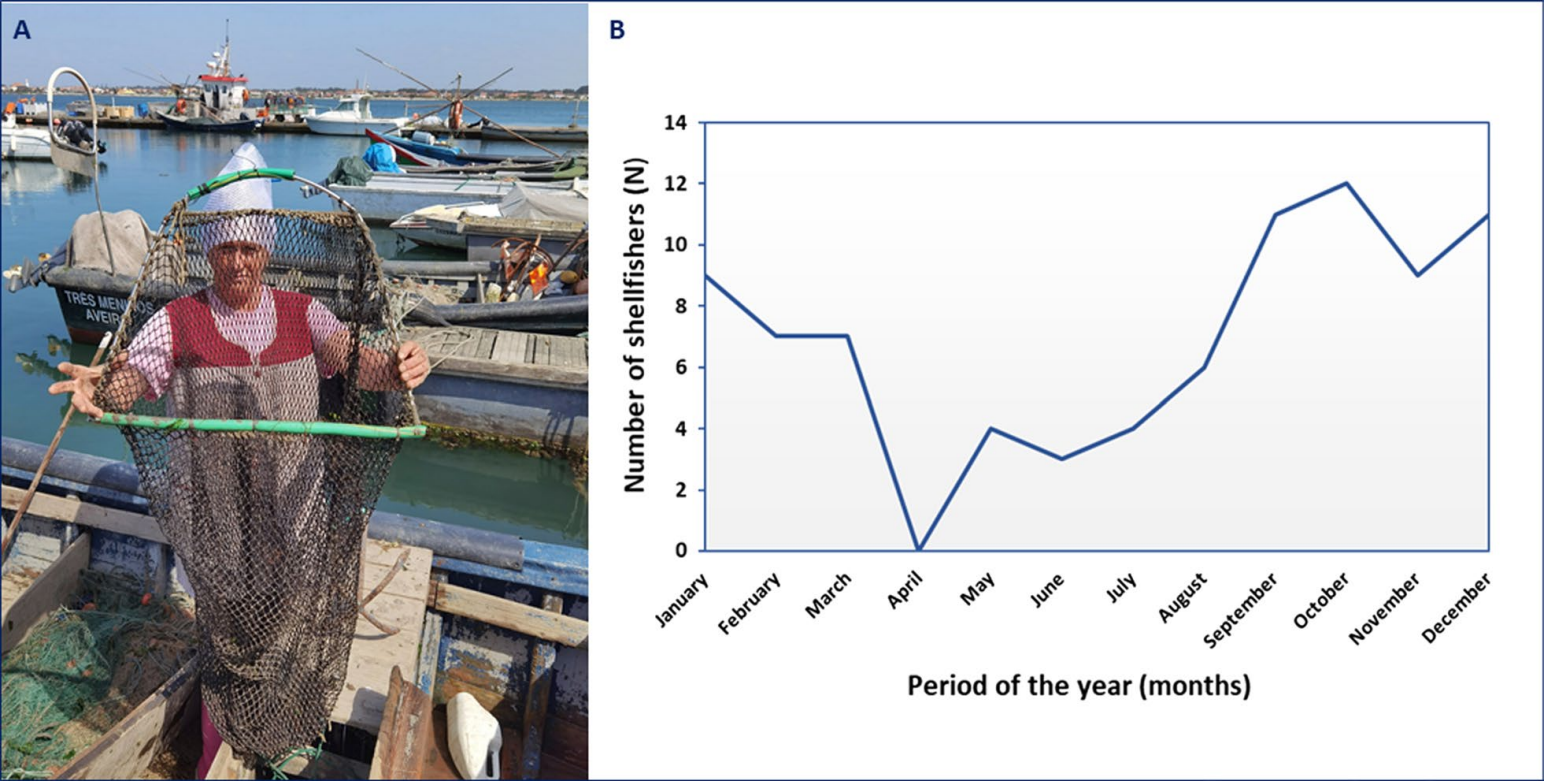
**A** Typical Shellfisher woman from the Ria de Aveiro coastal lagoon displaying a cockle harvesting tool locally called *nassa*. The photo was taken with prior authorization from the respondent, which is archived. *Source*: Braga, H.O. **B** Distribution of the cockle harvest over the months in the Ria de Aveiro lagoon, Portugal (*N* = 60)

We conducted 60 interviews (5 women and 55 men; Fig. 2) during April and May 2021 in the local communities of Aveiro—Murtosa (*N* = 13), Costa Nova (*N* = 15), Torreira (*N* = 16), and the Port of Aveiro (*N* = 16). The average age of the shellfishers was 51.07 years (minimum 21 years and maximum 82 years), with an average experience time of cockle harvesting of 29.33 years. Schooling was generally basic, with most respondents (*N* = 56) having elementary school (up to 9 years of study). The profile of shellfishers in Aveiro corresponds to the profile of shellfishers in Portugal [48–51]. Portuguese fishers have already shown themselves as a middle-aged workforce with a low level of education (concentrated in primary or preparatory education) [50]. Furthermore, according to other studies documented in Portugal [51], Aveiro’s shellfishers also had extensive experience in the fisheries sector.

The gathering of cockles in the coastal lagoon of Aveiro was carried out in an artisanal and traditional way. Fifty-three harvesters used small fishing boats to capture cockles. The boats used were the *bateira* (*N* = 32) and the fiber boats (also called “chata”, *N* = 19). The average fishing boat size was 6.56 m × 1.84 m. The average crew per trip was 3.04 shellfishers, and about 29 interviewees harvested the cockle alone or with a companion on board. The *bateira* has already been recognized as the most traditional fishing boat in Aveiro, being called *berbigoeira*, destined to catch cockles that typically had dimensions that could reach 13.8 m in length [52]. However, there was a tendency to use smaller boats up to 7 m in length (small *bateiras*) in the current fisheries in the Ria de Aveiro. The number of crew on fishing boats has not changed in recent decades, with an average of 3 fishers per boat in cockle fishing.

Shellfishermen mentioned five tools that were used to harvest cockles in the Ria de Aveiro (hand rake or *ancinho*—*N* = 52; *nassa*—*N* = 45; *joeira* or *ciranda*—*N* = 49; *cabrita*—*N* = 50; *ganchorra*—*N* = 11; Additional file 2 and Fig. 2). Three fishers picked cockles only manually. The most cited utensil in the Ria de Aveiro for the collection of cockles (hand rake (Additional file 2: Fig. S1A) consists of a wooden or metal bar, with teeth (straight or curved end of variable size, number, and spacing) fixed to a wooden or metal handle being used on foot and at low tide [53]. This fishing gear from Aveiro is a type of hand dredge [54]. The *nassa* (Fig. 2) has a conical or cylindrical shape and is dismountable, consisting of a small mesh net mounted on hoops or other rigid structures [54]. *Joeira* or *ciranda* (Additional file 2: Fig. S1B) is a type of sieve used to separate the permissible-sized cockles from small cockles. The *cabrita* or *berbigoeiro* (Additional file 2: Fig. S1C) is a hand dredger designed to capture cockles consisting of a metallic structure connected by a wooden handle with teeth whose size, spacing, and number are variable [53]. This fishing gear can be small *cabrita* (short handle) and large *cabrita* (long handle). *Ganchorra* (Additional file 2: Fig. S1D)*,* in this context, possibly referred to a type of towed dredger conducted by trawlers that operate on bottoms that are not discovered at low tide on the ocean coast [54]. In the Ria de Aveiro, the hand dredger (*ganchorra de mão*), also called a *berbigoeiro*, and hand and rake picking have already been reported as the main fishing gear for catching bivalves in the Ria de Aveiro [55].

According to 45 interviewees, cockle fishing was practiced every day of the week when an interruption of fishing was not imposed. These eventual obligatory interruptions occur when levels of marine toxin-producing phytoplankton or microbiological contaminants in the Ria de Aveiro exceed allowed values [56]. These periodic analyses are carried out by the National Monitoring System for Bivalve Molluscs (SNMB) of the Portuguese Institute for Sea and Atmosphere (IPMA) and aim to ensure the health control of bivalves intended for human consumption [32].

Cockle fishing was oriented according to the variation of the tides (*N* = 45). Six respondents indicated a preference for the morning period to collect cockles in the Ria de Aveiro, and three respondents preferred the afternoon period. All harvesters highlighted low tide as the preferred tide, and 36 respondents additionally mentioned mid-tide. The harvest time per tide ranged from 1 to 8 h (average of 4.16 ± 1.05 h). The minimum harvest size ranged from 16 to 60 mm. The mean minimum harvest size was 23.4 ± 7.2 mm. The minimum allowable capture size is 25 mm [57]. However, there were also shellfishers reporting the capture of individuals below 25 mm. The average size of this capture may also indicate that the cockle may be harvested before the first year of age [18]. In an estuarine area of Portugal, visible disturbances of the population structure of *C. edule* have been reported due to human overharvesting [58]. These bivalves caught below the allowed size may be accompanied by inadequate collection tools that can reduce the sustainability of cockle stocks and surrounding biodiversity in the Ria de Aveiro. [59].

The harvest per tide ranged from 0.5 kg to 700 kg, with an average of 137.12 ± 107.49 kg. The maximum daily catch limit for cockles per fishing boat duly licensed for fishing is 200 kg in the Ria de Aveiro [60]. A total daily catch limit of 50 kg of cockles per licensed fisher was also established by the Minister of Agriculture, Rural Development, and Fisheries of Portugal to ensure the sustainable exploitation of the resource [60]. The areas with the highest cockle harvesting effort in the Ria de Aveiro were RIAV1 (*N* = 53), RIAV2 (*N* = 43), RIAV3 (*N* = 30), and RIAV4 (*N* = 4). Fishers’ LEK is in line with data from the collective effort to distribute the bivalves target species of the Ria de Aveiro in 2012 [61]. Cale da Moacha and Cale do Ouro (RIAV1), and the Mira channel (RIAV2) were also the areas with the highest relative abundance of cockles in the Ria de Aveiro with about 70% of the total cockle biomass captured in 2013 [62].

The cockle harvest throughout the year showed how easy it is to catch this bivalve, with solid commercial demand and little investment in equipment and workforce [18]. The main harvest period lasted from September through March (Fig. 2). Cockles harvested were destined for export (*N* = 52), factories (*N* = 51), own consumption (*N* = 43), trade and food industry (*N* = 41), and bait for fishing (*N* = 6). The main export destination for cockles was Spain (*N* = 48). This Iberian country is one of the main target markets for shellfish exports from Portugal [63]. In Spain, these bivalves are destined for large and strong seafood canning industries [18]. This product sometimes returns to Portugal in frozen form and is sold in supermarket chains.

Respondents also mentioned that there were on average 683.67 ± 557.21 shellfishers along the entire length of the Ria de Aveiro. This figure is much higher than the 413 shellfishers (multispecific gatherer) registered by fishing associations in Aveiro (Data provided by the Fishing Associations in Aveiro). This finding shows how local authorities present obstacles in applying measures to control fishing efforts [18]. Some shellfish gatherers mentioned in the interviews may be related to illegal, retired, and not registered shellfishers with the local associations. The inadequate control of some areas of the Ria de Aveiro where bivalves are diverted and harvested without considering the minimum landing size [61] may be facilitating the continuation of unsustainable exploitation practices in this aquatic ecosystem. Most shellfish gatherers were registered with some fishing association in Aveiro (*N* = 44).

### Cockle ethnoecological knowledge

**Fig. 3:**
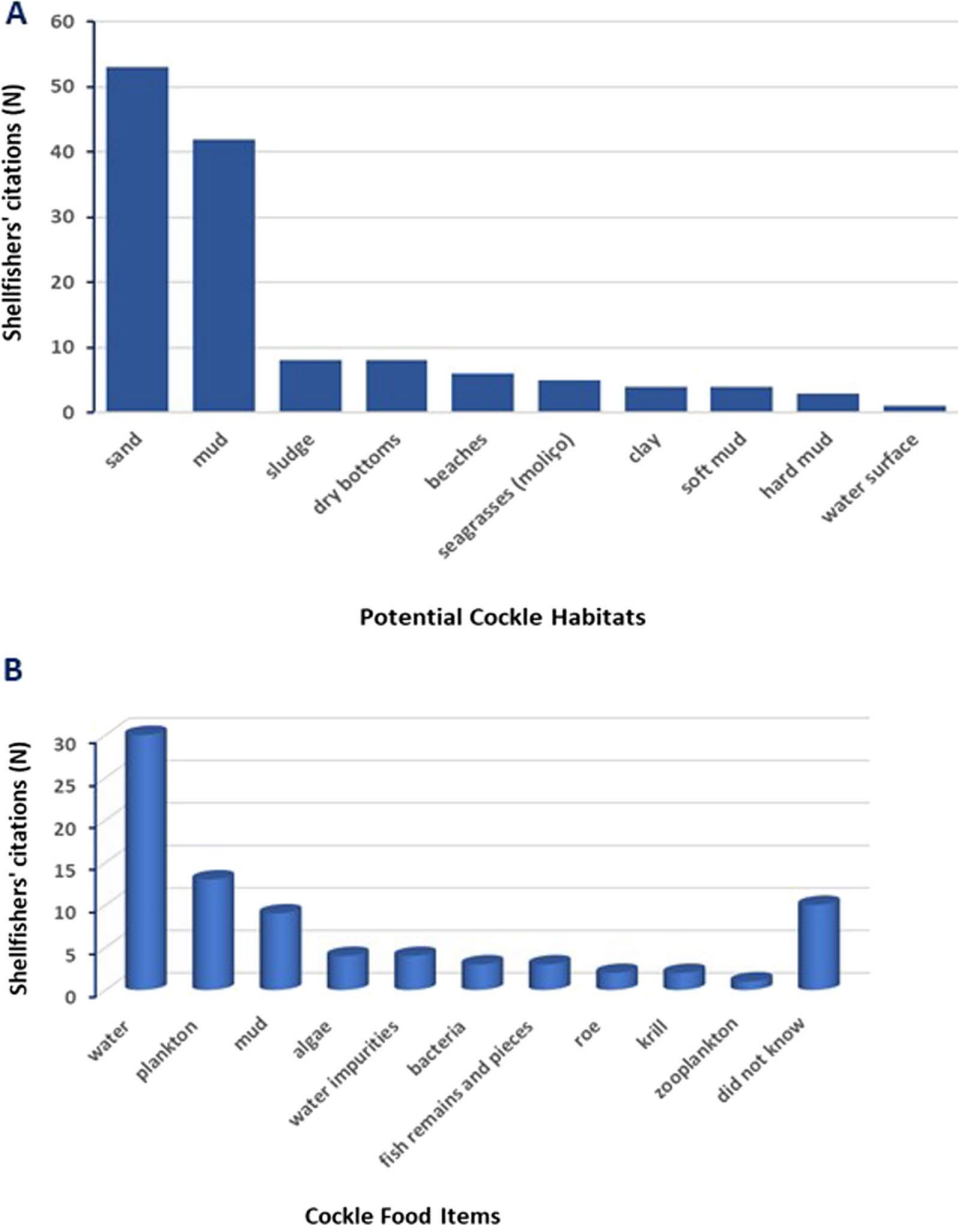
Ethnoecological knowledge about preferential habitats (**A**) and food items (**B**) of cockles in the Ria de Aveiro, Portugal (*N* = 60)

*Habitat* The Aveiro coastal lagoon presents varied natural values with numerous habitats for bivalves [35], such as the cockle. This bivalve can predominantly inhabit the first few centimeters of sediment zones [21]. Fishers’ LEK showed that the edible cockle *C. edule* could be found buried at a depth of 10 cm. The average depth was 2.71 ± 1.68 cm. The most frequent value of the LEK on cockle depth was 1 cm. Cockle sampling studies usually limit up to 10 cm in the sediment [64], which is within the range considered by shellfishers from Aveiro. The cockles’ preferred habitats (Fig. 3A) in the Aveiro lagoon were sand (*N* = 53), mud (*N*  = 42), sludge or muddy sand (*N* = 8), and dry bottoms (*N* = 8). Ecological studies also indicated that this bivalve lives in sediment surface, muddy sand, sandbank, mud gravel bottom and is found mostly in intertidal and subtidal areas [5, 18, 65, 66].

*Food items* This study shared a range of potential cockle food items (Fig. 3B). These food items were typical of bivalves suspension filter feeders with a fundamental role in purifying the water column, organic filtration, and energy flow in the biological community [5, 58, 66]. The main ethnobiological data about the foods consumed by cockles were: small particles present in water (*N* = 30), plankton (*N*= 13), mud (*N* = 9), algae (*N* = 4), water impurities (*N* = 46), bacteria (*N* = 3), fish remains and pieces (*N* = 3), roe and krill (*N* = 2 each), and zooplankton (*N* = 1). Some respondents did not present any ethnobiological data on this topic (*N* = 10). Cockles can feed on zooplankton, phytoplankton, organic particulate matter, juveniles of their own species, and eggs and larvae [58]. These bivalves still consume small particles suspended in the water column, including non-living materials such as suspended soil particles and plant debris [8].

*Predators* LEK provided twenty folk names of potential cockle predators in the Ria de Aveiro (Table 1). This finding reinforces the importance of cockles in the food chain as a link between primary producers and consumers [66]. The wide spectrum of cockle predators shared through the LEK highlights this critical potential in the ecological function of the ecosystem, especially regarding the influence of this bivalve at higher trophic levels [67]. This study highlighted the crabs—Probably: green crab—*Carcinus maenas* (Carcinidae) (*N* = 36). Some shellfishers (*N* = 10) specifically cited green crab. Shellfishers also cited the European plaice—*Pleuronectes platessa* Linnaeus, 1758 (*N* = 16), European eel—*Anguilla anguilla* (Linnaeus, 1758) (*N* = 15), seagulls (see Table 1) (*N* = 14), European seabass—*Dicentrarchus labrax* (Linnaeus, 1758) (*N* = 11), Great cormorant—*Phalacrocorax carbo* (Linnaeus, 1758) (*N* = 11) and Greater flamingo—*Phoenicopterus roseus* Pallas, 1811 (*N* = 11). We emphasize that shellfishers did not strictly say which stage of cockle development these living beings consume. They also did not say whether feeding in certain circumstances only occurs when the cockle is already broken in the environment. Cockle predation is also characterized by being very specific as it varies according to the size of this bivalve [8]. *Cerastoderma edule* is a crucial prey for demersal fish, birds, shrimps, and crabs [66, 67]. In our findings, there are reports of cockle consumption by fish species such as the European plaice and the crab *Carcinus maenas* [68]. Many wading birds with protection status are also cockle consumers [8]. The common eider (*Somateria mollissima*), oystercatcher (*Haematopus ostralegus*), and herring gull (*Larus argentatus*) are potential consumers of cockles [69]. Gastropods such as *Hexaplex trunculus* also present this bivalve as a food supply [70]. Forty-five shellfish gatherers said there were many cockle predators in this lagoon ecosystem, eleven said there were few predators, and eight said there was a moderate number of predators. The most abundant predator in the Ria de Aveiro was probably the foraging crabs of the Carcinidae family (green crab—*Carcinus maenas*; *N* = 8), and the least abundant was the European eel (*N* = 11).

**Table 1:** Main likely predators of the edible cockle *C. edule* in Ria de Aveiro, Portugal

Folk name (Portuguese)	Common name (English)	Scientific names (Linnaean)	Shellfishers’ citations and frequency (%)
*Caranguejos*	Crabs^a^	Crabs—probably: *Carcinus maenas* (Carcinidae)	(*N* = 36; 60%)
*Solha*	European plaice	*Pleuronectes platessa* Linnaeus, 1758	(*N* = 16; 27%)
*Enguia*	European eel	*Anguilla anguilla* (Linnaeus, 1758)	(*N* = 15; 25%)
*Gaivotas*	Seagulls	Seagulls in general—Probably: *Larus fuscus* Linnaeus, 1758 and *Larus michahellis* J.F. Naumann, 1840 and *Larus melanocephalus* Temminck, 1820	(*N* = 14; 23%)
*Robalo*	European Seabass	*Dicentrarchus labrax* (Linnaeus, 1758)	(*N* = 11; 18%)
*Corvo Marinho*	Great Cormorant	*Phalacrocorax carbo* (Linnaeus, 1758)	(*N* = 11; 18%)
*Flamingo*	Greater Flamingo	*Phoenicopterus roseus* Pallas, 1811	(*N* = 11; 18%)
*Linguado*	Common sole	*Solea solea* (Linnaeus, 1758)	(*N* = 8; 13%)
*Aves*	Birds	Birds in general	(*N* = 5, 8%)
*Peixes*	Fish	Fish in general	(*N* = 5, 8%)
*Garça*	Egret	Egret in general—Probably: *Egretta* *garzetta* (Linnaeus, 1766) and *Ardea cinerea* Linnaeus, 1758	(*N* = 4; 7%)
*Tainha*	Mullet	*Mugil* spp.	(*N* = 4; 7%)
*Cegonha-branca*	White stork	*Ciconia ciconia* (Linnaeus, 1758)	(*N* = 3; 5%)

^a^Within the group of crabs were specifically mentioned the green crab (*N* = 10; 16.7%)—*Carcinus maenas* (Linnaeus, 1758)

**Fig. 4:**
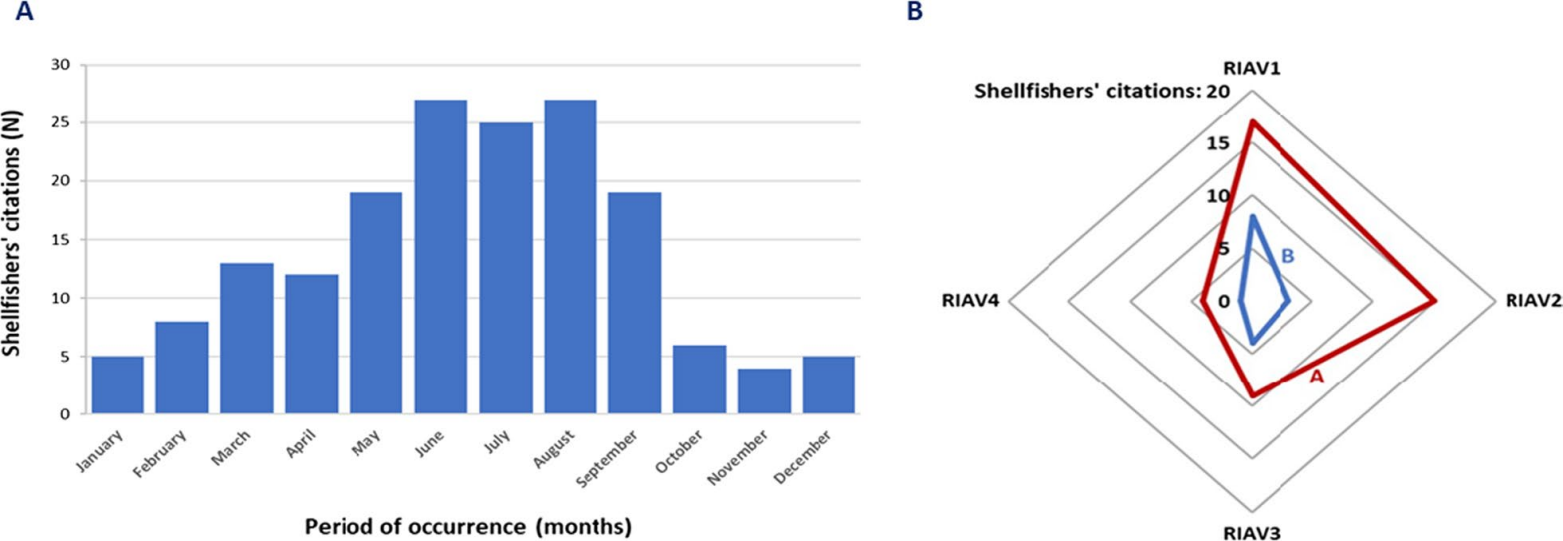
Cockle spawning period according to shellfishers (**A**) and Cockle harvesting effort by production zones in the last years at Ria de Aveiro, Portugal (**B**). In (**B**) **A** Decreased and **B** Increased

*Spawning *The edible cockle is considered a gonochoric species, even if hermaphrodite specimens or individuals with records of sexual reversals have been found [71, 72]*.* The *C. edule* spawning event took place throughout the year (Fig. 4A). The results mainly highlighted the end of spring (May and June) and the summer (July, August, and September). Gametogenesis of the main cockle species generally occurs between February and March, development of the gonads in April and May, and spawning between May and August [4]. Maia and collaborators (2021) reported that the spawning season for cockles in the Ria de Aveiro lagoon could occur from March to October, predominantly in the summer months (July to September) [18]. The spawning period reported in the present study is similar to that observed in this last biology investigation at Aveiro. On the European coast, findings equivalent to those in our shellfish LEK study have also been reported [4, 11, 73].

*Ethnoconservation of the cockle* Informants shared that the most recent decades were the greatest decrease in cockle stocks in the Ria de Aveiro lagoon (2000–2009: *N* = 7; 2010–2019: *N* = 20; 2020–2021: *N* = 7). In the late 2000s, published data for this bivalve species already registered a decrease in the biomass and abundance of cockles, highlighting the urgent need to improve the management of this fishery in Aveiro [61]. The main factors behind the decrease in cockles in the region, according to the interviews, were overharvesting (*N* = 13), increased rainfall in the lagoon (*N* = 6), and land modification (suction dredging, and the construction of a canal for tourism—*N* = 4). The high number of fishing boats, the increase in the variety of fishing gear (especially the trawl gear), the presence of the Pacific oyster—*Crassostrea gigas* and the Japanese carpet shell—*Ruditapes philippinarum* nurseries, and the pollution (agriculture and factories) were also remembered (*N* = 3). There were 45 quotes from shellfishers in which they indicated a perception of decreased cockles’ productivity (Fig. 4B) in RIAV1 (*N* = 17), RIAV2 (*N* = 15), RIAV3 (*N* = 9), and RIAV4 (*N* = 4). There were 16 quotes from shellfishers sharing information that there was an increase in cockle productivity in the RIAV1 (*N* =  8), RIAV2 (*N* = 3), RIAV3 (*N* = 4), and RIAV4 (*N* = 1) zones in the last years.

Burdon et al. [74] identified some factors that cause mortality in *C. edule*, such as food limitation, temperature and salinity, changes in sediments, suspended solids, topography and bathymetry, oxygen depletion, persistent depletion, toxic pollutants and organic loads, pathogens, parasites, and commensals. Variation of salinity gradients can affect estuarine organisms, especially in cases of abrupt changes [5]. Different responses of estuarine organisms to salt stress may be related to structures and differences in habitat at each stage of development [14]. Fishers’ LEK showed that the increase in freshwater in estuarine environments due to rainfall events might be related to the decrease in cockle productivity in some production zones of the Aveiro lagoon. According to Verdelhos and collaborators [5], *C. edule*’s population structure could be substantially altered in the face of extreme climatic events such as floods.

The action of suction dredging in specific fishing grounds can remove the larger cockles from the tidal plains and generate mortality of other fauna, making the habitat unsuitable for some species [75]. Effects of disruption of the bottom may also be collaborating to impact certain production zones in Aveiro. The bivalve harvesting method should also be considered a control measure for the possible indirect effects of fishing exploitation [76]. Bivalve harvesters in Ria de Aveiro had already called for more effective control of bivalve dredgers towed from boats, as this method of trawling affects fishery resources and the entire aquatic ecosystem [61] and is illegal. Shellfishers cited a variety of harvesting methods carried out in Ria de Aveiro. However, according to the interviewees, the capture of cockles by boats through trawling stood out as a possible threat to cockle stocks. Improving the management of the capture methods used in the Ria de Aveiro in the intertidal and subtidal zones is essential since it becomes a possible solution for the conservation and more selective and efficient exploitation of this bivalve [18].

The conservation status indicated that the cockle population in the Ria de Aveiro is stable (*N* = 27). Other respondents said that the species was threatened (*N* = 14), highly threatened (*N* = 9), not threatened (*N* = 7), and little threatened (*N* = 3). *Cerastoderma edule* has not yet been evaluated for the International Union for Conservation of Nature (IUCN) Red List, and the European Nature Information System (EUNIS) species database does not provide information on the conservation of this species [77, 78]. Aveiro’s shellfishers also shared some harvesting possibilities to conserve the cockle in their natural territory in the Ria de Aveiro. This LEK on the proper harvesting of the cockle is described in Table 2.

**Table 2:** Shellfishers’ recommendation for a proper cockle harvest (*N* = 60)

Shellfishers’ LEK about harvesting in the habitat	No. of citations
*“Cockle harvesting must be done with proper fishing gear and tools and the correct sizes.”*	33
*“Trawl gear must not be used when harvesting cockles.”*	29
*“You must collect and leave the small cockles in the same terrain where they were caught.”*	25
*“It will help if you spread out the remaining cockles that are on the ground. It would be best if you did not leave them huddled on the floor.”*	09
*“Casulo*^**a**^ *should not be caught in the cockles’ habitat.”*	07
*“It should only have manual cockle harvesting.”*	02

^a^“Casulo” refers to the *Diopatra neapolitana* Delle Chiaje, 1841 annelid species belonging to the Onuphidae family

Shellfishers reported that the constant presence of researchers in the Ria de Aveiro and the interaction with local fishing villages could favor the conservation of the cockle. Forty-five interviewees said they tended to accept scientific advice from university researchers about cockle harvesting and cockle’ conservation in the Ria de Aveiro. Some respondents (*N* = 13) remained neutral in the face of this question. Only two informants did not want to give an opinion. These findings showed that some members of the Aveiro community of cockle harvesters were predisposed to collaborate to conserve the cockles. The involvement of these fishing villages in collaborative management with all active stakeholders can favor the underlying patterns and allow for the testing of monitoring tools to improve results in marine systems [79].

Some shellfishermen (*N* = 6) question the effectiveness of analyzing chemical contaminants and biotoxins carried out by government technicians periodically in the Ria de Aveiro. In this coastal lagoon, there is a plan to monitor and detect various toxins in areas to ensure public health and safe trade for seafood consumers in Portugal and exporting countries [80]. The main shellfishers’ concerns about this point were the lack of transparency in collecting samples, the care taken with transporting these samples to the place where analyses were carried out, and the distrust about the points in the lagoon where the technicians collect the samples. Promoting alternative environmental education activities with the fishing community to publicize existing procedures and standards can improve trust among all interested parties [81]. Even with this type of action through discussion and education, some fishers may still have attitudes contrary to the established norms [82]. However, understanding and analyzing the knowledge of artisanal fishers become crucial for a more flexible approach to the conservation of fisheries resources in these communities dependent on biological resources [83].

## Conclusion

This present research broadly showed the local ecological knowledge (LEK) provided by shellfishers about the edible cockle (*C. edule*) in the Ria de Aveiro. Shellfishers provided a cumulative body of knowledge in line with several previously published data on the biology and ecology of edible cockles [4, 5, 8, 11, 18, 21, 35, 58, 64–70, 73]. This LEK emerges as an essential auxiliary tool to mutually benefit conservation biologists and the local population [83, 84]. It tries to create a sense of ownership over the bioresource conservation to resource users and an opportunity for them to collaborate in a more cooperative debate about local sustainability [85]. We showed that well-designed and applied interviews in a reliable and ethical environment can generate reliable information on bioresources. Considering the LEK in future adaptive management processes to better understand how these local communities respond to the uncertainties and unpredictability of natural resource population dynamics through social learning [86].

Given the continuous and growing exploitation of bivalves in the Ria de Aveiro, socio-ecological strategies become necessary. Additional studies on gaps in the ecology of *C. edule* in the Ria de Aveiro may add even more value to the future creation of the management plan for this bivalve in this coastal zone. Unrefuted LEK data from this study should not essentially be discarded. Managers can analyze and verify this information to recognize the values of all interested parties linked to the fishery resource in question. Some unrefuted hypotheses can still be explored in biological investigations. Given the multispecific fisheries in the Ria de Aveiro [18], ethnobiological studies of other targeted species may add even more information from artisanal fishing in favor of sociocultural and comprehensive conservation biodiversity in Ria de Aveiro, Portugal.

## Supplementary Information

**Additional file 1**. Semi-structured Interview Script.

**Additional file 2**. Illustrations of the tools used by the shellfishers to harvest cockles in the Ria de Aveiro.

## Data Availability

Not applicable.

## Abbreviations

TEK: Traditional ecological knowledge
LEK: Local ecological knowledge
ISE: International Society of Ethnobiology
SPA: Special protection area
APARA: Association of Artisanal Fishing of the Region of Aveiro
EUNIS: The European Nature Information System
IUCN: The International Union for Conservation of Nature
GBIF: Global Biodiversity Information Facility
Fish Base: Global information system on fishes
