# Supplementary material for: A case study of local ecological knowledge of shellfishers about edible cockle (Cerastoderma edule) in the Ria de Aveiro lagoon, Western Iberia
Source: J Ethnobiol Ethnomed. 2022 Mar 5;18:11. doi: 10.1186/s13002-022-00507-x (PMC8897764; doi:10.1186/s13002-022-00507-x)
Supplement: Supplementary file 1 — Additional file 1. Semi-structured Interview Script. [file 13002_2022_507_MOESM1_ESM.docx]

**Supplementary Information**

***Additional file 1:* Semi-structured Interview Script.**

**_____________________________________________________________________________________**

***Part I. Shellfishers’ Profile.***

**- Date: - Interview N^o^.: - Local: - Age: _______. - Gender: ( ) Male ( ) Female.**

**- Education: ( ) A: Low level (illiterate and elementary education 1 - up to 5 years of study). ( ) B: Basic level (Primary education 2 - 6 to 9 years). ( ) C: Intermediate level (High school - 10 to 12 years). ( ) D: Advanced Level (13 years or more).**

**- Fishing experience (years)?**

**_____________________________________________________________________________________**

***Part II.*** ***Shellfishers’ knowledge about cockle fishing.***

**- What kind of fishing do you practice?**

**- Do you use boats? What type and size (m x m)? What is the maximum number of the crew?**

**- What tools and equipment do you use to catch cockles?**

**- How many times do you catch cockles a week?**

**- What is the average time to catch cockles per tide?**

**- What is the minimum size of the cockle you catch?**

**- What is the amount collected per tide (kg or bags)?**

**- Where do you usually practice this cockle catch (specific areas of the Ria de Aveiro or RIAV)?**

**- What are the months of cockle picking in the Ria de Aveiro?**

**- What is the final destination of these cockles captured from Ria?**

**- How many shellfishers should be dedicated to collecting cockles in the Aveiro estuary (on average)?**

**- Are you registered in any association of shellfisheries? What is the name?**

**_____________________________________________________________________________________**

***Part III. Local ecological knowledge (CEL) of the cockle (Cerastoderma edule) in the Ria de Aveiro.***

**- At what maximum surface depth (cm) does this species live buried?**

**- What are the main types of locations (habitats/funds) preferred by the cockle?**

**- What does the cockle consume (eat)?**

**- What are the main natural predators (animals that eat) of the cockle?**

**- Are there a lot of these predators in the Ria de Aveiro?**

**- What is the time (months) of cockles spawning in the Ria de Aveiro?**

**- After which decade did you notice a decrease in the quality and success of cockle capture (quantity) in the Ria de Aveiro? Why?**

**- How should cockle catch be carried out (excavation) so that you can always have cockles in the Ria de Aveiro (conservation)? Report care.**

**- What is your position regarding the advice from researchers from Universities about cockle catching and conservation in the Ria de Aveiro? Why?**
