# Supplementary material for: A case study of local ecological knowledge of shellfishers about edible cockle (Cerastoderma edule) in the Ria de Aveiro lagoon, Western Iberia
Source: J Ethnobiol Ethnomed. 2022 Mar 5;18:11. doi: 10.1186/s13002-022-00507-x (PMC8897764; doi:10.1186/s13002-022-00507-x)
Supplement: Supplementary file 2 — Additional file 2. Illustrations of the tools used by the shellfishers to harvest cockles in the Ria de Aveiro. [file 13002_2022_507_MOESM2_ESM.docx]

**Supplementary Information**

***Additional file 2:* Illustrations of the tools used by the shellfishers to harvest cockles in the Ria de Aveiro.**


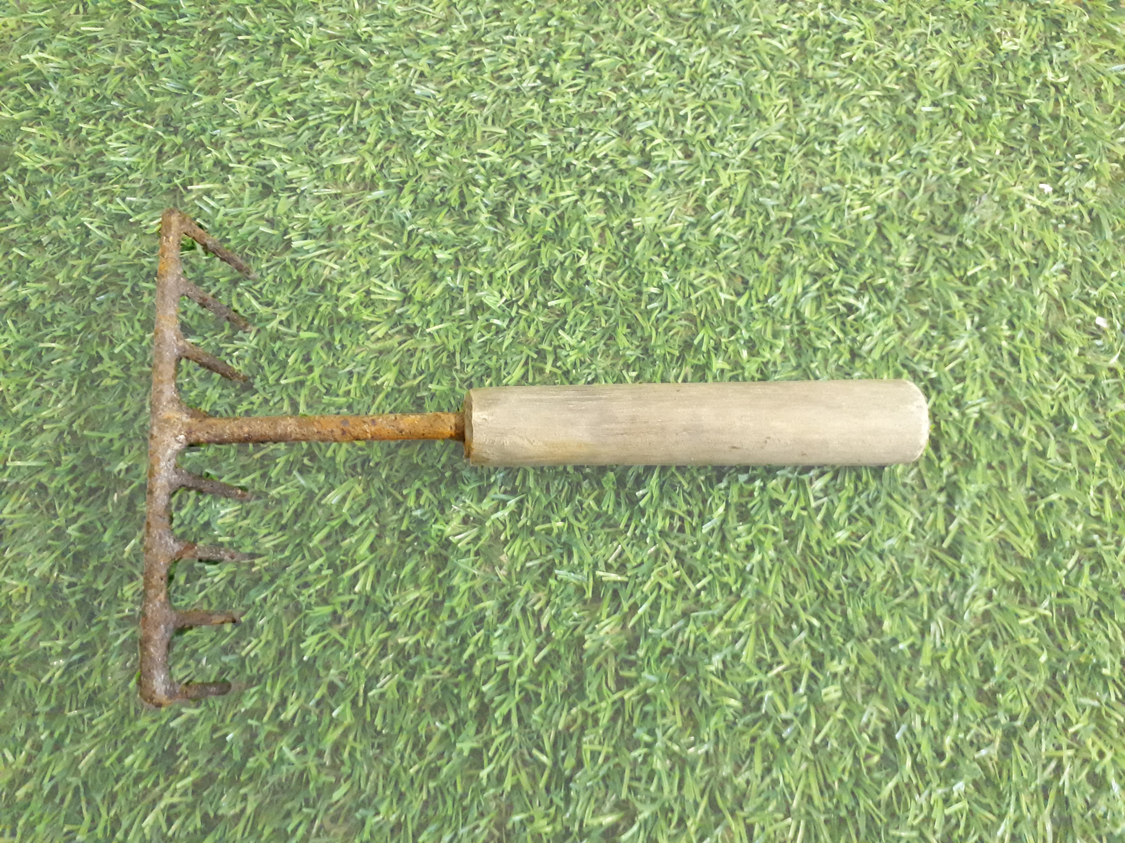

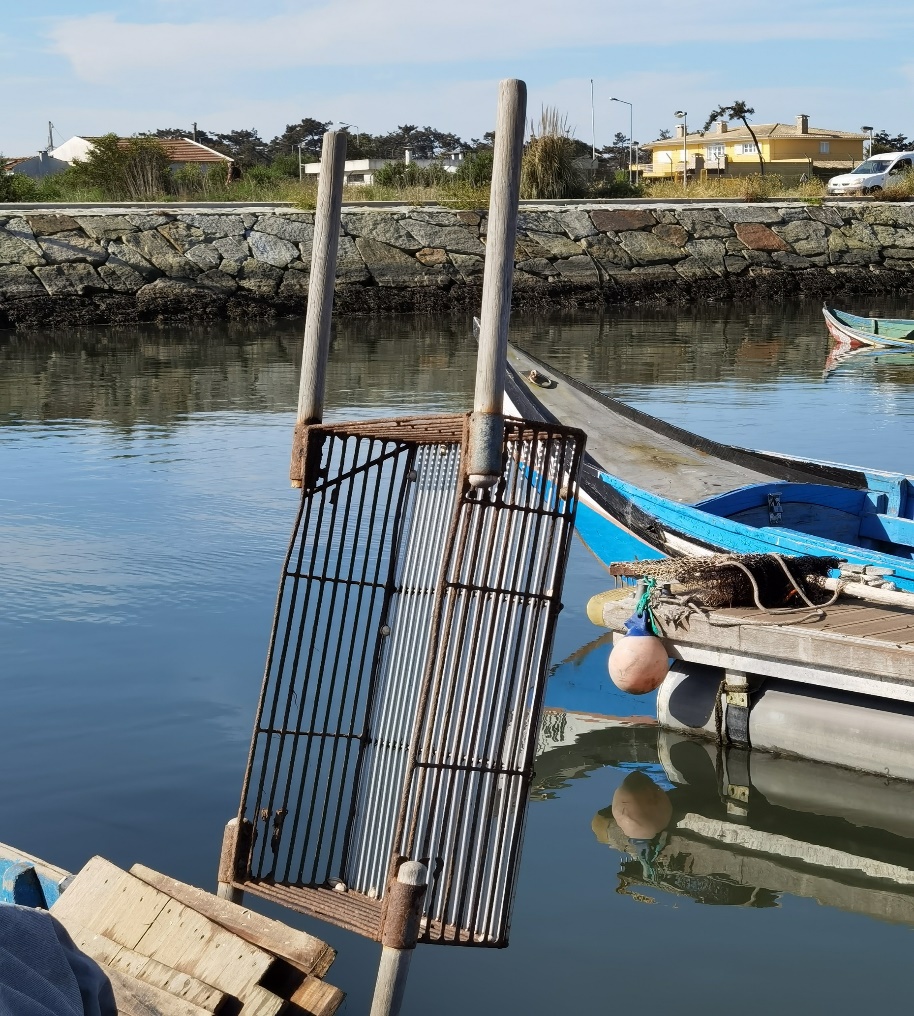

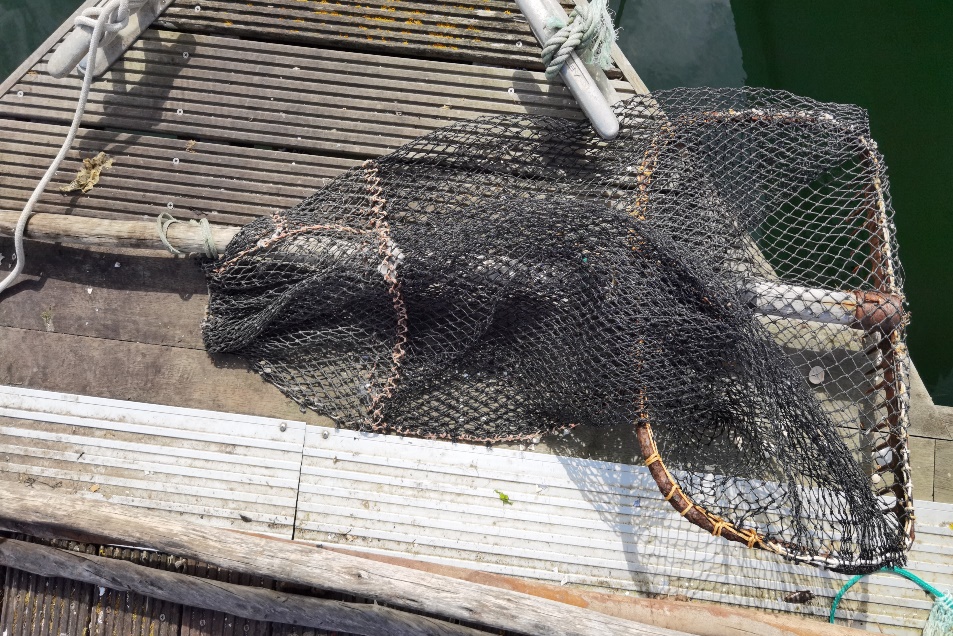

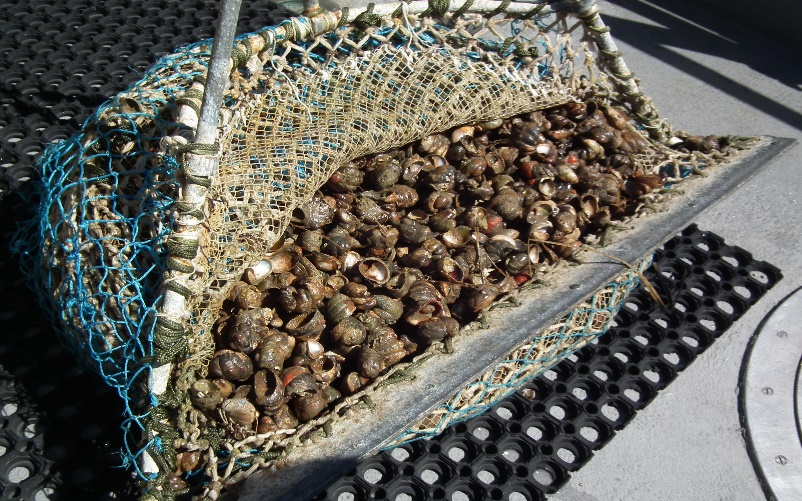


**A**

**B**

**C**

**D**

**Figure 1.** ***A:*** Hand rake or *ancinho*. Source: Magalhães, L. ***B:*** *Joeira* or *ciranda*. Source: Braga, H.O. ***C:*** *cabrita* or *berbigoeiro*. Source: Braga, H.O. ***D:*** *ganchorra*. Source: Magalhães, L.
